# Factors Affecting the Immunity to Respiratory Syncytial Virus: From Epigenetics to Microbiome

**DOI:** 10.3389/fimmu.2018.00226

**Published:** 2018-02-19

**Authors:** Wendy Fonseca, Nicholas W. Lukacs, Catherine Ptaschinski

**Affiliations:** ^1^Department of Pathology, University of Michigan, Ann Arbor, MI, United States; ^2^University of Michigan, Mary H. Weiser Food Allergy Center, Ann Arbor, MI, United States

**Keywords:** respiratory syncytial virus, neonatal immunity, epigenetics, microbiome, metabolites

## Abstract

Respiratory syncytial virus (RSV) is a common pathogen that infects virtually all children by 2 years of age and is the leading cause of hospitalization of infants worldwide. While most children experience mild symptoms, some children progress to severe lower respiratory tract infection. Those children with severe disease have a much higher risk of developing childhood wheezing later in life. Many risk factors are known to result in exacerbated disease, including premature birth and early age of RSV infection, when the immune system is relatively immature. The development of the immune system before and after birth may be altered by several extrinsic and intrinsic factors that could lead to severe disease predisposition in children who do not exhibit any currently known risk factors. Recently, the role of the microbiome and the resulting metabolite profile has been an area of intense study in the development of lung disease, including viral infection and asthma. This review explores both known risk factors that can lead to severe RSV-induced disease as well as emerging topics in the development of immunity to RSV and the long-term consequences of severe infection.

## Current Status of RSV Disease and Treatment

Respiratory syncytial virus (RSV) is an omnipresent virus that infects nearly all children by age 2 years (1). It is also the most common cause of hospitalization in children under 2 years of age, causing severe lower respiratory tract infection, bronchiolitis, and pneumonia, and is associated with an increased risk of developing childhood asthma and recurrent wheezing (1, 2). Severe RSV infections often exhibit an inefficient activation of innate immunity and skewing of the acquired immune response toward a Th2 and/or Th17 phenotype leading to airway mucus overproduction, with both an excessive Th2 response and with IL-17 highly upregulated in infants (1, 3–6).

Considerable effort has been expended to develop an effective vaccine against RSV infection without success. The prophylactic use of a neutralizing monoclonal antibody against the RSV F envelope protein (palivizumab) prevents progression of RSV severity in the high-risk patient with RSV infection (7). It is also used as a therapy to control the infection in patients who present with RSV-related bronchiolitis (7, 8). However, this treatment is expensive, and most infants who develop severe infection do not exhibit risk factors, necessitating a better understanding of what drives severe disease in order to develop new therapeutics or effective vaccines.

**Figure 1:**
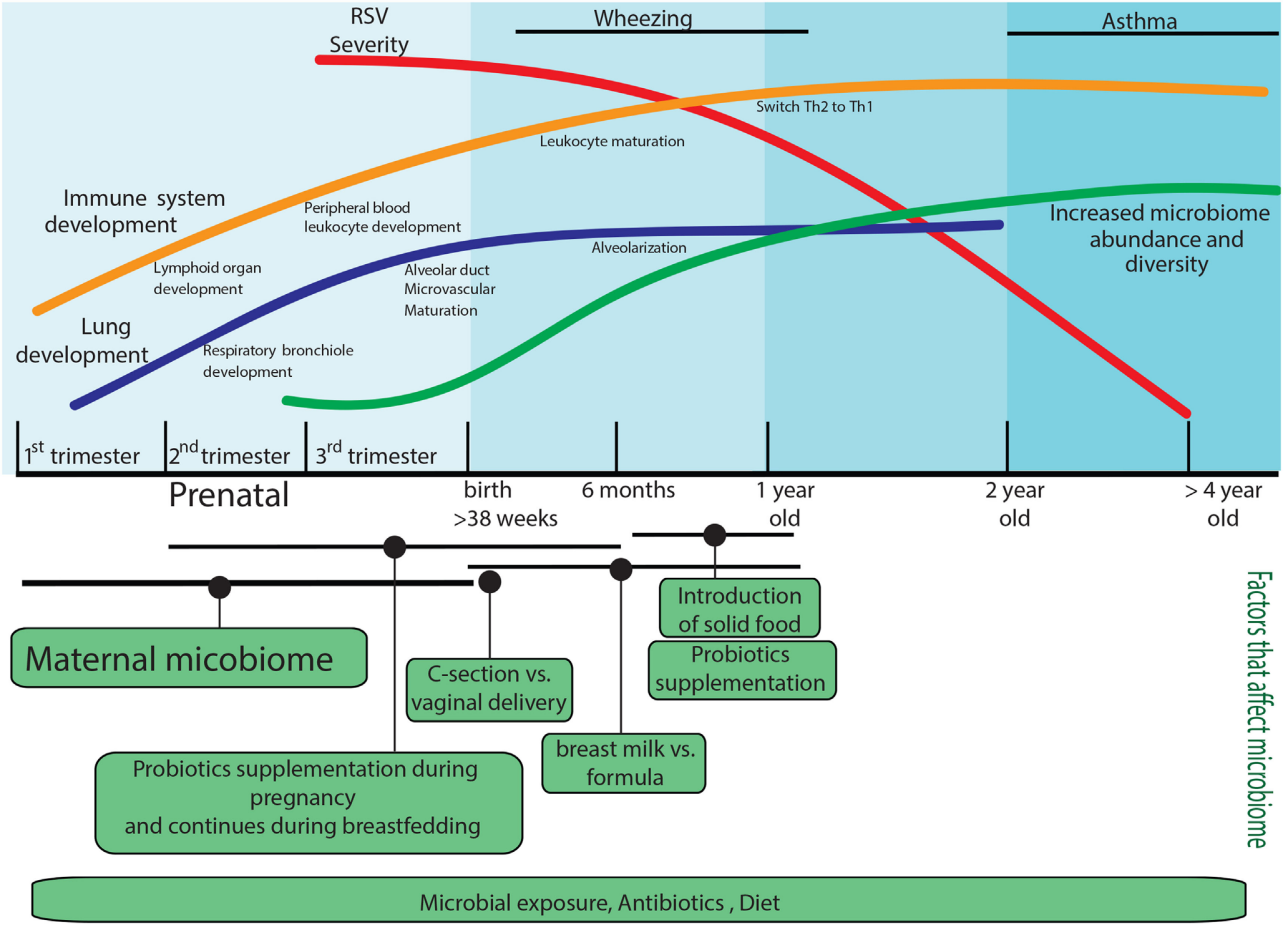
Factors that predispose to the development of severe respiratory syncytial virus (RSV) disease. Factors that can impact the immune response during RSV infection are age; lung development is not complete in preterm infants, and the immune system development will continue until the first years of life, and it will be impacted by the microbiome composition of the mother and infant. The infant microbiome would be shape since the prenatal stage by the mother microbiome, and it will continue modifying by postnatal factors such as environmental microbial exposure, mode of delivery, diet, and antibiotic use. The infant microbiome would have long-acting effects on RSV immune responses. All these components independently or together represent the most common elements that are involved in the development of RSV severe disease.

In this review, we cover several aspects that contribute to the immune response to RSV, including the development of the immune system and the lung environment, the role of epigenetics in this development, and the contribution of the microbiome to long-term pulmonary health. These factors are summarized in Figure 1.

## The Neonatal Immune System and RSV

The age at which a child is infected with RSV plays a critical role in disease outcome. Infants who are infected before 6 months of age are at higher risk for developing bronchiolitis that requires hospitalization, even when compared with bronchiolitis caused by other viral infections (9). One of the primary reasons for this is that the neonatal immune system has a number of functional differences when compared with the immune response of adults.

Early in life, immune protection is mediated primarily by the innate immune system, as adaptive immunity does not begin to develop until after birth and continues developing through early childhood (10). Furthermore, the immune system is skewed away from a pro-inflammatory state, as this can lead to spontaneous abortion and damage to the developing tissue, especially the lungs (11). Thus, neonatal immune cells exhibit low levels of pro-inflammatory cytokines including the type I interferons (IFN), IFN-α and IFN-β, as well as low levels of IL-12 and TNF-α, but higher levels of anti-inflammatory cytokines such as IL-10 and TGF-β (12–14).

Because the neonatal system is reliant on innate immune recognition, signaling through toll-like receptors (TLRs) is relatively well developed in neonates. These pattern recognition receptors are a critical component of the innate sensing of pathogens in infants. However, although TLR sensing and signaling in infants occurs on a similar level to that in adult cells, the immune mediators that result from these signaling pathways are impaired in infants. While type I IFN levels rise rapidly in the first month of life, IL-12, which is important for driving a Th1 response, is one of the last cytokines to reach adult-like levels (13, 14). This increase in pro-inflammatory cytokines is mirrored by a decrease in anti-inflammatory IL-10 following TLR signaling (13). IL-6 and IL-23 are also enhanced in TLR-stimulated neonatal cells compared to adults (13, 14). As these cytokines are potent inducers of IL-17, neonates have an increase in Th17 cells (15). Because increased IL-17 is often associated with enhanced RSV-induced pathology, the prevalence of this cytokine in infants may contribute to enhanced disease (3, 4, 6). An additional level of TLR regulation in neonates is the high level of adenosine found in the plasma of umbilical cord blood (16). Adenosine is a metabolite that is released in response to inflammatory events and acts as a feedback mechanism to decrease TLR-dependent Th1 inflammation (17–19). This combination of both cell-intrinsic factors such as decreased cytokine production and exogenous factors such as plasma adenosine limits the function of TLRs in the neonates.

Dendritic cells (DCs) express a number of TLRs that are essential for dictating T cells responses based on the ability of DCs to present antigen to T cells causing their activation, and subsequent cytokine production. Myeloid DCs (mDCs) are the primary source of the IL-12 needed to drive a Th1 response. As mentioned above, IL-12 levels are reduced in neonates compared to adults. mDCs are also a significant source of other pro-inflammatory cytokines including IFN-β, which is robust in adult cells but impaired in neonatal DCs (20, 21). Neonatal and adult mDCs also exhibit a number of other differences including lower expression on neonatal cells of MHC-II for antigen presentation and CD80/86 for costimulation of T cells (21, 22). Activation through TLR signaling or cytokine addition fails to upregulate these surface molecules to a level seen in adult mDCs (21). Plasmacytoid DCs (pDCs) are another subset of DCs involved in directing inflammatory responses. While in adults, the number of mDCs is higher than pDCs, neonates have approximately three times the amount of pDCs as mDCs (23). However, even with this increased predominance, pDCs from neonates fail to adequately mature upon stimulation, including decreased production of type I IFNs that are critical for the antiviral response (12, 24).

Neutrophils are another critical innate cell type, which function independently of antigen recognition. While neutrophil infiltration into the lungs in response to RSV may contribute to tissue damage, these cells also help limit the spread of the virus and participate in the induction of the adaptive immune response (25–28). Infants have a higher number of neutrophils in the blood than adults, but these cells have decreased function (29). Neutrophils can control viral infection through phagocytosis and release of cytotoxic granules for killing the infected cells; both of these features are impaired in neonatal neutrophils (29, 30). During RSV infection, neutrophils can phagocytose RSV-infected cells as well as protect epithelial cells from infection, providing a protective role against viral replication (25, 31, 32). Although damaging effects of neutrophils are thought to contribute to a decline in lung function following infection, they are an essential part of the innate immune response to RSV (28).

Natural killer (NK) cells are one of the more prominent cells types in the blood of neonates, with levels exceeding those of adult blood. However, like many other cell types, the function of these cells is drastically reduced early in life. NK cells bind to target cells, such as virally infected cells, and release cytotoxic granules to kill the infected cells. The cytotoxic ability of neonatal NK cells is decreased compared to adults, as cells from infants have fewer cytotoxic granules as well as a decreased capacity for degranulation to release their contents (33, 34). Whether these cells have dysfunctional migration is unknown; however, infants with severe RSV infection have been noted to have low numbers of NK cells, underscoring the need for these cells in a productive response to the infection (35). NK cells also produce IFN-γ, and neonatal NK cells produce less of this Th1 cytokine than adult cells (36). However, unlike other cell types, these defects are not intrinsic to neonatal NK cells but are instead due to decreased signaling and activation from pro-inflammatory cytokines made by other immune cells. Exogenous addition of IL-12 to neonatal NK cells restores their cytotoxic effects and ability to produce IFN-γ (36–38). As mentioned above, IL-12 production from DCs is delayed in neonates, thereby resulting in an environment that is less permissive for NK cell activation, as well as Th1 cell differentiation.

Neonatal CD4^+^ T cells are skewed away from a pro-inflammatory Th1 response and instead favor a Th2 or Th17 response, both of which have been implicated in severe RSV infection (4, 39). Like NK cells, this is at least in part due to extrinsic factors such as decreased DC-derived IL-12 in the environment, as these cells can produce IFN-γ when transferred into an adult environment (40). However, there are T cell-intrinsic factors that limit Th1 responses as well, including a number of epigenetic factors that will be described later in this review. Additionally, many of the circulating T cells in neonates are known as recent thymic emigrants, which do not proliferate as well as other circulating T cell subsets and produce lower levels of IFN-γ (41). Finally, neonates have increased numbers of regulatory T cells (Tregs), which limit inflammatory responses early in life (42–44). As previously noted, this is important for preventing tissue damage. In addition, maternal immune cells can cross the placenta and reside in developing fetal lymph nodes. As some of these cells can recognize proteins derived from paternal genes, Tregs are a critical mechanism for suppressing this maternal immunity and preventing fetal rejection (45).

The decrease in CD4^+^ T cells in neonates translates to altered B cell function early in life. As these T helper cells are needed to drive antibody production from B cells, neonates have delayed antibody responses compared to adults, including antibody responses to RSV (46, 47). Neonates also have a different distribution of IgG isotypes, with lower levels of Th1-associated IgG2 (48). Antibody responses in neonates are also of lower titer, lower affinity, and persist for less time, but begin to reach adult-like function after 6 months of age (49–51). In general, the primary antibody protection in infants comes from maternal antibody transfer, both *in utero* and through breast milk following birth. This latter aspect has led to the support of developing a vaccine for RSV that would target mothers in order to transfer maternal protective antibodies to infants to provide early life protection from severe infections (52–54).

## Preterm Birth and RSV Risk

As previously mentioned, infants under 6 months of age are at a higher risk of severe RSV infection requiring hospitalization for RSV-induced bronchiolitis. This is especially true of infants who are born prematurely (<37 weeks gestation) (55, 56). There are number of reasons why premature birth results in increased risk for severe RSV infection. The differences in the immune response between infants and adults are more pronounced in preterm neonates. As mentioned above, maternal antibodies are a significant source of immune protection in neonates and are able to cross the placenta during pregnancy, and the majority of this occurs during the third trimester (57). Babies born to mothers with high levels of RSV neutralizing antibody are protected from severe diseases. However, this protection is to an extent lacking in preterm infants (58). Thus, treating preterm infants with palivizumab has become more standard for these infants born within RSV season, which occurs during the winter months (58).

As noted earlier, neutrophils are critical regulators of innate immunity and are especially important in early life when the adaptive immune response has not fully developed. However, neutrophils in preterm neonates exhibit reduced function compared with those from term infants. It has long been known that neutrophils from preterm neonates have decreased migratory ability compared with the cells from term neonates (59, 60). Neutrophils from these infants release lower levels of bactericidal/permeability-increasing protein than cells from term infants, which may result in increased susceptibility to infection (61). Recently, it has been shown that neutrophils from preterm infants have decreased pathogen recognition and antimicrobial ability as well (62). One study found that nasal washes from preterm infants have fewer leukocytes and lower levels of IL-8 than washes from term infants, which is a key cytokine in the recruitment of neutrophils, indicating a role for neutrophils in RSV infection of preterm neonates (63).

Numerous other innate immune defenses are also deficient in preterm neonates compared to term neonates. A small study of infected infants found that preterm infants infected with RSV had fewer pDCs in the bronchoalveolar lavage fluid than those born at term (64). In addition to decreased cell number in preterm infants, monocyte activity is also decreased. A recent study found decreased inflammatory gene expression in monocytes of preterm cord blood compared with both term cord blood and adult peripheral blood (65). Upon exposure to RSV, lowere IL-6 production was noted in monocytes from the cord blood of preterm infants compared to term and adult blood (66). The overall reduction in the innate immune cell responsiveness to RSV infection may be intrinsic and therefore difficult to overcome by vaccination, especially if muted adjuvant reactivity is also a lingering issue (66).

In addition to immunologic deficiencies in preterm neonates compared to those born at term, complications also arise due to differences in lung development. The initial capacity for oxygen exchange occurs late in development when alveolar septal formation begins at approximately 34 weeks of gestation (67, 68). This increases the number of alveoli in the distal airways, thereby increasing the surface area available for oxygen exchange. As this process continues into the postnatal period, premature birth before the process is fully underway can have serious consequences. The primary pathology that occurs in the lungs from premature birth is bronchopulmonary dysplasia (BPD), which results in a decrease in the number of alveoli (69). RSV infection of infants with BPD results in a much higher rate of hospitalization than for RSV-infected infants without BPD, even when comparing infants born at similar gestational ages (70). Treatment of these patients with appropriate anti-viral therapy or palivizumab, although complex, would greatly reduce the hospitalization rates due to RSV infection (70).

## Epigenetic Modifications Affecting the Immune Response to RSV

**Figure 2:**
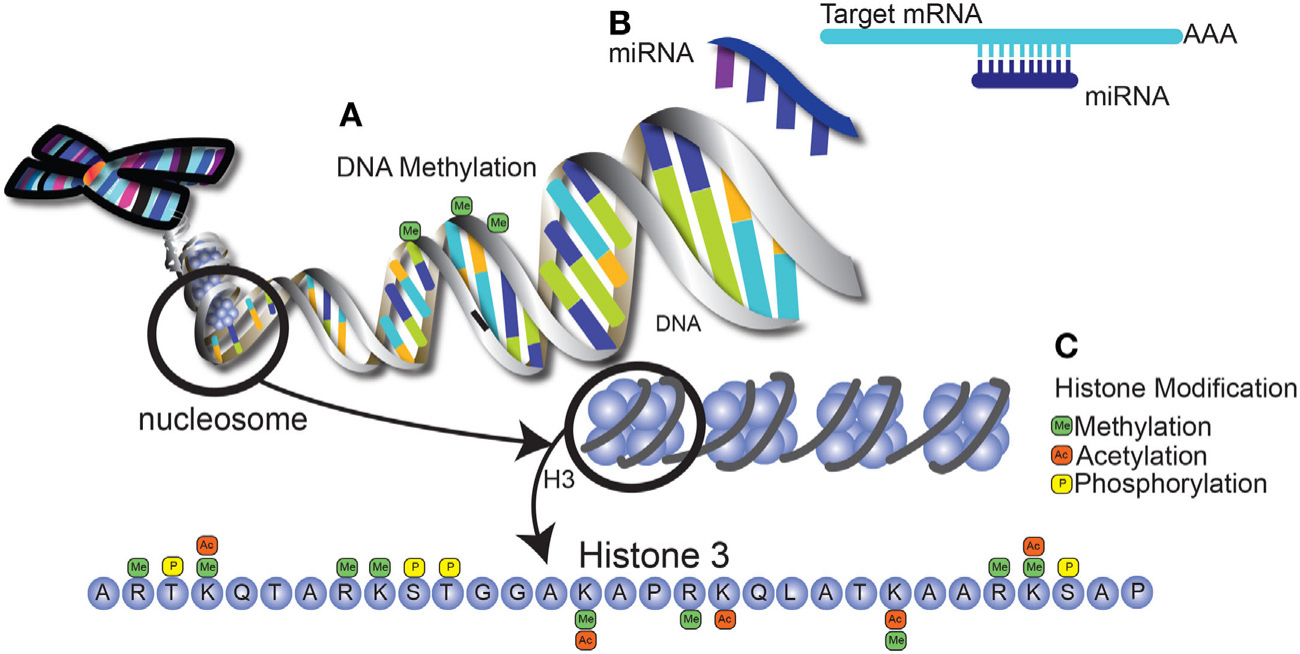
Epigenetic modification alters gene expression through multiple mechansims. **(A)** DNA methylation occurs at regions that are rich in cytosine and guanine (CpG islands), leading to the repression of gene transcription. **(B)** MicroRNAs are short, non-coding RNA molecules encoded in the genome that bind to mRNA, leading to mRNA degradation or translational suppression. These matches can either be fully complementary or may contain mismatched bases, allowing one miRNA to target several mRNA molecules. **(C)** Histone modifications include methylation and acetylation of lysine and arginine residues on histone tails, as well as phosphorylation of serine and threonine residues. Potential modifications of the tail of histone 3 are shown as an example. These modifications result in changes in chromatin structure that can be either repressive or permissive for transcription factor binding.

Many of the differences in immune function between neonates and adults are due to epigenetic modification of genes that control inflammation. Epigenetics refers to mechanisms that alter gene expression without changes to the sequence of underlying DNA, thereby controlling many aspects of development. The three primary types of epigenetic regulation are histone modifications, DNA methylation, and microRNA expression. Histone modifications can regulate gene expression by altering residues on the histone tails, such as methylation, acetylation, and phosphorylation. In general, histone modifications "fine tune" the gene expression by altering chromatin structure. This change in structure can wind the chromatin more tightly to prevent access of transcription factors and repress gene transcription. Alternatively, the change in chromatin structure can result in increased DNA accessibility to transcription factor binding, therefore promoting gene activation. These modifications are referred to as “activation marks” or “inhibitory marks.” For example, methylation of lysine (K) 27 of histone (H) 3 (i.e., H3K27) is associated with repression of gene transcription, whereas H3K4 methylation is permissive for active gene transcription (71–73). This process is orchestrated by methyltransferases that catalyze the addition of methyl groups and demethylases that remove them. Other modifications such as acetylation and phosphorylation have similar mechanisms of action. On the other hand, DNA methylation results in the inhibition of gene transcription, resulting in genes being turned “off” (74–76). This primarily occurs on cysteine residues in regions rich in cytosine and guanidine, known as CpG islands, in which methylation of the cysteine residues results in suppression of gene transcription (77). Finally, microRNAs are small non-coding RNAs that bind to messenger RNA. miRNA recognition of target mRNA is not always 100% complementary, so a single miRNA can target multiple mRNAs (78). miRNA interaction with mRNA results in either mRNA degradation (in the case of complementary recognition) or translational repression (in the presence of base pair mismatches), but both with the final result of inhibiting protein synthesis (79, 80). Together, these mechanisms are able to alter gene expression on multiple levels, as summarized in Figure 2.

### Epigenetic Regulation of Immune Cell Development

Several recent studies suggest that epigenetic modulation is important in dictating immune cell phenotype and function and allows the immune environment and/or the external environment to influence the outcome of the immune response. For example, IL-4, IL-5, and IL-13, which are produced by Th2 cells, are silenced in Th1 cells through histone modification (81). Conversely, Th17 cells have increased levels of the activating mark H3K4 methylation at the Th17 promoter (82). In DCs, decreased H3K4 methylation at the IL-12 promoter has been measured following severe sepsis, thereby altering subsequent immune responses and leading to higher susceptibility to subsequent infections (83). The epigenetic alteration of immune cells allows a more stable phenotype to develop in response to the environment, however sometimes in negative contexts (83).

In the neonatal immune system, further epigenetic regulation is present and prepares the immune cells for subsequent responses. As discussed above, neonatal immune cells are poised toward Th2 effector function. There are a number of epigenetic mechanisms behind this Th2 bias. Many studies have identified differential DNA methylation patterns in human neonatal cells compared to that of adults. In differentiated adult Th2 cells, the Th2 locus is hypomethylated, leading to a more permissive transcriptional state of Th2 cytokines (84, 85). In neonatal naïve CD4^+^ T cells, a similar hypomethylation is observed, indicating that these cells are poised for a Th2 response even before activation (86). Hypomethylation of the *IL13* distal promoter has also been observed in naïve human neonatal CD4^+^ T cells (87). Increased methylation of Th2 genes begins in the first year of life and continues out to 5 years of age (88, 89). Conversely, naïve human neonatal CD4^+^ T cells have increased CpG methylation at the *IFNG* promoter compared with adult cells, which correlates with decreased IFN-γ production (90, 91). Furthermore, hypermethylation has been observed at numerous Th1-associated genes in neonatal mononuclear cells compared with adults, indicating increased regulation of Th1 responses in neonates (92). These phenotypes must be actively altered to allow a more balanced response to occur, including moving the responses to environmental and infectious agents toward a more appropriate, nonpathogenic response (92).

MicroRNAs contribute another level of regulation of immune development early in life. Many miRNAs have been found to be differentially regulated in human cord blood samples compared to adult bone marrow or peripheral blood samples (93, 94). For example, monocytes isolated from cord blood express more miR-146a following LPS stimulation compared to cells from adult peripheral blood (95). As miR-146a is a negative regulator of TLR4 and increased expression of this miRNA results in a decrease in TLR4 responsiveness in neonatal monocytes, this prevents a robust pro-inflammatory response to bacterial infection (96). T cells are also differentially regulated by miRNAs in neonates. In neonatal CD8^+^ T cells, two miRNAs, miR-29 and miR-130, drive cells to become short-lived effector cells instead of memory effector cells, thereby altering the immunological memory to an antigen (97). Similarly, cord blood CD4^+^ T cells express higher levels of miR-181a and miR-184 compared to adult peripheral blood cells, which decrease maturation and activation of these cells (98, 99).

Fewer studies have identified changes in histone modifications in neonatal immune cells, although some work has been done in monocytes. In particular, altered nucleosome remodeling in neonatal monocytes has been linked with defective DC development and impaired IL-12 production (100, 101). More recently, an in-depth analysis of H3K4 methylation on neonatal monocytes was performed (65). Increased methylation of H3K4 is associated with increased gene transcription, and cord blood monocytes have dramatically lower levels of H3K4 methylation compared with adult cells. Furthermore, it was noted that H3K4 methylation is nearly absent in preterm neonates and is even lower than cord blood from term infants, indicating that DNA methylation is continuously increasing during development and that monocytes from premature infants are not "poised" for an appropriate innate immune response (65).

### Epigenetic Regulation by Environmental Exposures

There are a number of epigenetically regulated factors that may affect the severity of RSV infection in infants. Environmental exposure both *in utero* and following birth results in alterations of the lung environment that may be detrimental to developing the appropriate response to RSV infection. One of the best-studied exposures is that of tobacco smoke. Infants who are exposed to tobacco smoke *in utero* are more likely to be admitted to the intensive care unit with bronchiolitis (102). This risk is further increased when these infants continue to be exposed to tobacco smoke following birth (102). A number of studies have shown that *in utero* smoke exposure results in detrimental epigenetic changes. Prenatal exposure has been shown to alter the DNA methylation status of a number of genes into childhood (103). In particular, differential methylation of genes involved in T cell development has been observed in infants exposed to tobacco smoke prenatally compared with those that were not (104). A recent study has also identified miRNAs that are altered in the lungs of mice exposed to prenatal tobacco smoke, including miRNAs that regulate immune pathways (105).

Maternal diet is known to alter the epigenetic profile *in utero*. In particular, women are encouraged to take a folic acid supplement during the first trimester of pregnancy to prevent neural tube defects. However, folate also acts as a methyl donor, and children born to women who begin folic acid supplementation 6 months before pregnancy and continue supplementation throughout pregnancy have increased methylation at CpG islands in cord blood DNA (106). These children have an increased incidence of wheezing during childhood, as well as increased risk of hospitalization with respiratory infection early in life (107, 108). These early studies set up new paradigms to focus on the role of epigenetic changes that have long-term effects on the development of lung pathology.

### Epigenetic Modifications in Response to RSV

A number of studies have shown that inflammatory stimuli can alter histone methylation of immune cells, including influenza and sepsis (83, 109). We have previously found that RSV infection of DCs leads to alterations in histone methylation by the H3K4 demethylase KDM5B, resulting in decreased pro-inflammatory cytokine production and a subsequent increase of Th2 cytokines from T cells (110). We have also found that methylation of H3K4 in regulatory T cells by the histone methyltransferase SMYD3 is necessary to control inflammation in the lungs following RSV infection (111). Another group recently identified that IFN-γ stimulation of epithelial cells resulted in changes in H3K9 methylation status of the RIG-I promoter, thereby upregulating RIG-I (112). As RIG-I is an important component of the immune response to RSV, this resulted in decreasing viral load (112, 113). Other histone modifications induced by RSV infection include histone acetylation. Infection of bronchial epithelial cells results in increased histone deacetylase 2 (HDAC2) that appears to alter innate immune responses; inhibition of HDAC2 results in increased production of type I IFN and decreased viral replication both in cultured cells and following RSV infection of mice (114).

Respiratory syncytial virus infection has also been shown to alter DNA methylation status, as shown by a number of recent studies. In humans, cultured bronchial epithelial cells infected with RSV upregulate Nodal, a member of the TGF-β superfamily, resulting in increased Th2 and Th17 skewing of T cells (115). A study in the cord blood of children found that those who had increased methylation in the enhancer region of the perforin-1 gene had increased risk of developing lower viral respiratory tract infection, including an increased risk of RSV infection (116). Perforin-1 is an essential cytotoxic protein in CD8^+^ T cells and NK cells in the control of viral infection. A follow-up study found that children at 3–4 years of age who had been hospitalized with severe RSV-induced bronchiolitis as infants had decreased methylation of the perforin-1 enhancer, indicating a role for RSV in altering the immune response even years after infection (117).

Studies in mouse models suggest that RSV can alter miRNA expression in the airway and affect how the tissue responds to future stimuli, especially in the development of post-viral allergic asthma. Infection of allergic mice with RSV results in the development of steroid-insensitive allergic airway disease, and this event is driven by the upregulation of a miRNA that inhibits steroid sensitivity (118). Another model using pneumonia virus of mice (PVM, a rodent-specific RSV homolog) found that neonatal infection changed the expression of a set of miRNAs that altered the way the mice responded to allergen challenge as adults (119). The regulation of how these miRNAs are expressed and what cell populations are expressing the specific miRNAs is not clear, but this regulation likely dictates how they alter the subsequent allergic responses (119). Together, these studies demonstrate the many ways in which the immune system is regulated during RSV infection and the potential development of allergic asthma.

## Microbiome, RSV, and Asthma

As previously described, a number of studies have linked early severe RSV infection and the consequent RSV-bronchiolitis immunopathology to the development of chronic allergic diseases like asthma in the later years of life (120, 121). We have discussed some factors that contribute to the development of severe RSV disease such as prematurity (immature immune system), congenital conditions like bronchopulmonary dysplasia, and age (from premature infants to 6 months old). There are many additional known factors that predispose to develop severe RSV disease, including Cesarean (C)-section delivery and breastfeeding for less than 1 month, which are factors that are also known to affect the composition of the microbiome and to contribute to the development of allergic disease (122–124). The microbiome assembles during the first year of life and critically sets the characteristics of the adult microbiome, as well as contributes to healthy immune responses and metabolic processes in infants (124–126).

Our lab and others have shown that microbiome composition can impact the development of RSV pathogenesis and allergic asthma in mice previously exposed to enriched microbial suspension or probiotic prior to RSV infection or allergen challenge (127, 128). In this section, we will discuss the impact of the infant microbiome in the development of allergies and severe lower respiratory tract infection (LRTI).

### Infant Microbiome and the Development of the Immune System

The human microbiome has been shown to be involved in several essential processes in humans, from organ development to modulation and fine tuning of the immune system. The microbiome is formed by microbial communities, collectively termed as microbiota. Their composition is in constant change during life, with variations of the microbiota in a number of environments, such as the lung, gut, or skin. Several factors affect the diversity of the microbial communities: the use of pharmaceuticals, nourishment, microbial exposure, and infection. Changes in the microbial diversity can impact the general homeostasis of the organism (122–126).

### Impact of Prenatal Microbiome: Respiratory Virus and Allergies

Historically, the uterus has been thought to be a sterile environment, although studies have questioned whether there is microbial exposure during the prenatal period. Microbes have been described in the placenta, amniotic fluid, fetal membrane, umbilical cord blood, and meconium (129, 130). The possibility of the impact of this microbial community to the development of the immune system, metabolite production, and fetus health, in general, is an active area of investigation for many groups (129, 131–134). For example, in a study where pregnant mothers received probiotic supplements, a microbial alteration was detected in infant meconium compared to controls (130). Others have found that changes in microbiome during pregnancy are linked to different outcomes in the offspring from behavior alterations to allergy predisposition (130, 135).

Atopic dermatitis is one of the first and most studied diseases in infants used to predict their allergy susceptibility and the early manifestation of atopic dermatitis-like eczema has been associated with an increased risk of asthma (136, 137). A double-binding study showed that administration of probiotics to mothers during the last trimester of pregnancy and during lactation increased the immune-protective potential for the breast milk, by increasing TGF-b2, which is a known immunoregulatory factor, in the milk compared with placebo-receiving mothers. In this case, the infants from probiotic treated mothers had a significantly reduced risk of developing eczema during the first years of life compared with the children from placebo-treated mothers (130, 132).

Increased microbial exposure has been shown to modify the microbiome (127, 131). In a longitudinal study, a cohort of infants was followed from birth to 2 years of age and IgE was measured in blood samples. Infants born to mothers who owned pets (parental indoor pet exposure) had a lower titers of total systemic IgE from 6 months old than the children lacking prenatal pet exposure and the difference was significantly stronger at 2 years of age (134). In a double-blind trial, mothers from families with allergic disease received probiotic supplements in the last trimester of pregnancy. After birth, their babies continued with supplementation until 1 year old, and these babies had decreased allergic incidence compared to control groups. Specifically, these infants had less eczema associated with IgE-associated at 2 years of age and reduced risk of developing later respiratory allergic disease (136). Another longitudinal study found that infants who were at risk of developing asthma had a transient disruption of the gut microbiome during the first 100 days of life, with significant decreases of *Lachnospira, Veillonella, Faecalibacterium*, and *Rothia* genera (138). The reduction of these bacterial taxa also impacted the levels of acetate in the feces, as well as dysregulation of liver metabolites. Germ-free mice inoculated with these four bacteria showed decreased airway inflammation demonstrating the importance and impact of the early microbiome composition in preventing the development of asthma and possibly other allergic diseases in children (138). Together, the microbiome during pregnancy has been proposed as a critical component for the overall health of the infant. A healthy microbiome during pregnancy may modify microbial communities in the mother’s gut, vagina, and uterus and would transfer the healthy microbiome in gut, lung, and skin to the fetus/infant *in utero* and during vaginal delivery. Evidence suggests that this is correlated with a healthy microbiome in the infant.

### Mother to Offspring: Microbiome Alteration

It has been widely described that the mode of delivery strongly affects the microbiome species found in the neonates, specifically that C-section delivery increases the risk of several health conditions in the newborn including increases in the likelihood of respiratory distress even at term (139, 140). In addition, C-section deliveries are associated with an increased risk of developing asthma and gastrointestinal diseases, among others (141). Industrialized countries have experienced a rise in the rate of Cesarean delivery in the 20 last years, correlated with an increase of autoimmune diseases and also allergic disease including asthma, eczema, atopic dermatitis, and food allergies (142, 143). The implication of the microbiome modification by C-section and the impact in the early and adult life of the offspring to develop asthma is currently an area under investigation.

Using 16S rRNA sequencing to define the bacterial microbiota in stool specimens of a birth cohort collected during the first year of life, one group evaluated the associations between pre- and post-natal environmental and sociocultural factors and the composition of the early life gut microbiome. This study found substantial age-related taxonomic variation in the microbiota (144). At birth, the primary bacterial communities were *Bifidobacteriaceae* or *Enterobacteriaceae* taxa, while infants (<1-year olds) were typically dominated by either *Bifidobacteriaceae* or *Lachnospiraceae*, the latter of which represents a typical dominant family in adult gut microbiomes (144). The impact of delivery mode in the composition of the infant microbiome has been previously described, with infants delivered by C-section presenting significant different diversity in the gut microbiome compared with vaginally delivered infants (145). Besides compositional variation by mode of delivery overall (vaginal vs. C-section), significant differences were observed whether the C-section was planned or unplanned, indicating that even early labor may affect the microbial composition of the infant (139).

In a population-based national register cohort study, the effects of acute or elective C-section vs. vaginal delivery in children from 0 to 23 months of age were assessed. Hospitalization for RSV disease, with adjustment for external factors such as birthrate and maternal smoking during pregnancy, showed a positive association between delivery by C-section and increased risk of hospitalization for RSV infection, with this effect continuing out to 2 years of age (123). Together, these results suggest a correlation between RSV hospitalization and microbiome diversity modification by mode of delivery, although more research is needed in this area.

### Breastfeeding: Microbiome, Respiratory Virus, and Allergies

The microbiome and immune system are actively modulated by specific factors within breast milk, including IgA that confers passive immunity. Breast milk also includes several metabolites that can impact metabolism, development, and maturation of the immune system in the neonates (146, 147). As we described above, the neonatal immune system is not entirely mature during the first months of life, and thus the interaction with the different organisms, foreign proteins and chemicals will set the basis for a successful interaction between the innate and adaptive immune systems. The infant’s interaction with components of the commensal bacteria population helps to stimulate the maturation of the immune system. A critical component of the immune development driven by the microbiome is immune suppression at the gut mucosal surface, which induces oral tolerance to food antigens (145). Breast milk is known to contribute commensal bacteria to the infant gut microbiome, and several studies have shown that breast milk contains approximately 10^3^–10^4^ colony forming units per milliliter, thus being an excellent source of probiotics (147).

A number of studies have investigated the link between breastfeeding and the development of respiratory disease. In a prospective cohort study, newborns were followed up for 1 year to monitor hospitalization for bronchiolitis (148). These infants were classified as “never breastfed” and “ever breastfed,” with the second group further divided into those “exclusively breastfed” and “breastfed associated with milk formula.” The risk of hospitalization at 1 year of age for bronchiolitis was significantly higher in the “never breastfed” group. Infants who were exclusively breastfed or breastfed associated with formula milk demonstrated a similar, reduced risk of bronchiolitis-related hospitalization, thus confirming the protective effect of maternal milk in the development of severe bronchiolitis (148).

In another study, infants who tested positive for RSV were divided into full, partial, or never breastfed as above, and were followed for 10 days after the initial diagnosis to analyze the incidence and duration of hospitalization and the frequency of requiring oxygen therapy. While there were no differences in the hospitalization rate among the three groups, those infants who were never breastfed had an increase in the duration of hospital stay and required oxygen therapy at a greater rate than the breastfed groups (149). This study agrees with another cohort of infants (<1-year olds) hospitalized for bronchiolitis, which found that the lack of breastfeeding correlated with higher rates of bronchiolitis (150).

We describe above that the prenatal microbiome composition impacts the development of allergies in the offspring and that probiotic supplementation infants received from their breastfeeding mothers decreased the risk of atopic dermatitis (146), indicating that the microbiome composition of the mother during breastfeeding also impacts the immune system of the infants. It is known that human milk composition can be modulated by the maternal environment and also that breast milk is unique between mothers (146). These data suggest that probiotic supplementation by the mother during breast feeding could be a best strategy aimed at decreasing further the incidence of allergy development and LRTI (RSV) in breastfed infants.

### Microbiome Modification in Infants: Supplementations and Microbial Exposure

In addition to probiotic supplementation of pregnant women, supplementation of children has also been studied. These studies show a similar effect to those observed in mothers on probiotic supplements during both pregnancy and breastfeeding, reinforcing the idea of the gut microbiome shaping the immune response to asthma and LRTI. The lung microbiome has also been studied. A shift in the microbial composition of the lung during respiratory diseases suggests a link to the host immune response to viral infection (151). An experimental study in mice that were exposed nasally to two different strains of *Lactobacillus rhamnosus* showed that the intranasal administration of these commensal bacteria modulated the TLR3/RIG-I antiviral respiratory immune response, where both strains demonstrated increased resistance of infant mice to RSV infection compared to controls (152). Another study in which young mice (3 weeks old) were orally supplemented with *Lactobacillus rhamnosus* CRL1505 before a RSV challenge showed improved resistance against RSV infection with decreased immunopathology, along with altered expression of IFN-γ and IL-10 in bronchoalveolar lavage, suggesting that the modulation of the immune response by probiotics early in life could favor protective immunity against RSV (153).

In humans, a randomized study with preterm infants was grouped to receive oral probiotics (*Lactobacillus Rhamnosus* GG) or placebo, between days 3 and 60 of life. These infants were followed to assess the effects on viral respiratory tract infection. The results showed a significant decrease in the detection of rhinovirus in nasal swabs in the children that were treated with probiotic compared with the placebo group, suggesting that the supplementation of probiotics in premature infants may prevent rhinovirus infection, although there were no significant differences in the detection of RSV (154). Prenatal characteristics were described but not analyzed (154), and as mentioned above, the prenatal microbiome regime likely modified the immune response of the infants positively (130, 132). These results suggest that the supplementation in infants after birth may not be as an effective as prenatal intervention.

Household microbial exposure during the first years of life can shape the microbiome of children, and it has been suggested that the increased microbial exposure during the prenatal stage and the early years of live affect maturation of the immune system and help to reduce the risk for the development of allergic diseases (155). The protective effect of allergic diseases by microbial interaction with farm animals and pets during early life has been studied by a number of groups (155, 156). In a cross-sectional survey in rural areas in Europe, children from farming families who were exposed younger than 1 year to farm animals had significantly decreased allergy sensitization with lower frequencies of asthma, hay fever, and atopic sensitization (155).

In another study demonstrating the importance of microbial exposure in early life, a birth cohort study of healthy, full-term infants were followed until the age of 6–7 years. These children had a significantly decreased risk of atopy when exposed during the first year of life to two or more dogs or cats, suggesting that local household microbial exposure in the early years of life confers protection against allergic disease development (156). Following these studies, the same group determined whether the presence of pets in the household increased the microbial communities and if this microbial material conferred protection (126). They examined the diversity of bacterial and fungal communities in dust collected from the homes with pets and observed significant enrichment of bacterial communities in the dust samples from houses with pets compared to the dust from houses without pets. A vast majority of the enriched bacteria found were also detected in the human gut microbiome, suggesting that the exposure to enriched microbial communities conferred a protective effect on the development of allergies (126, 156). Thus, it appears that multiple factors during pregnancy and early infancy impact the development of the microbiome, which can shape immune function throughout childhood.

## Microbial Metabolites and Their Role in RSV and Asthma

The gut microbiota is necessary for proper food digestion, and a number of metabolites are a result of digestion by these bacteria. Several studies support the concept that airway immune status is impacted by the metabolite profile produced by the gastrointestinal microbiome (128, 157, 158). The metabolites from intestinal microbiota are the result of the microbial-host interactions, which are a complex system dependent on the food intake of the host, the external environment, the use of antibiotics, and the other microbial genera occupying a person’s microbiome. Together, these interactions generate a host of metabolites that can impact the immune response systemically and locally in organs of the host, including the lung and gut (128, 157, 159).

Using animal models, the effect of the dietary fermentable fiber content has been studied in the composition of the lung and gut microbiota (157). In one study, mice were raised on a diet with standard, low or high fiber, followed by intranasal exposure to house mite extract. The mice that had low fiber diet showed increased inflammatory cell infiltration and increased Th2 cytokines in the lung, with elevated total IgE serum levels compared with the control, standard diet. The authors also observed that DC isolated from the lung of the low fiber diet mice exhibited a more active phenotype, as determined by increased CD40 and CD80 (157). Furthermore, the mice that were fed high fiber diets demonstrated reduced inflammatory cell infiltration and decreased Th2 cytokines in the lung, as well decreased levels of total IgE. The isolated lung DCs were also less activated, with reduced expression of CD40 and CD80. Finally, the authors observed that increasing the fiber in the diet modified the composition of the microbiota, with a difference in the ratio of *Firmicutes* to *Bacteroidetes*. The gut microbiota degraded the fiber and increased the concentration of systemic short-chain fatty acids (SCFAs). Increased circulating levels of SCFAs were observed in mice fed a high fiber diet, resulting in protection from allergic airway inflammation. Conversely, mice fed a low fiber diet had decreased circulating SCFAs, correlating to increased allergic disease in the lungs (157). Supplementing mice with the SCFA propionate altered hematopoiesis and the phenotype of the DCs. The authors concluded that fermentable dietary fiber and SCFAs could modify the immunological environment in the airway and influence the severity of allergic inflammation (157). In a human study, bacteria were grown from the intestinal microbiota of infants who were at high risk of atopic diseases, followed by gas-liquid chromatography to detected bacterial cellular fatty acids (158). The authors found that the bacterial cellular fatty acid profile in fecal samples was significantly different between atopic and healthy infants, and that the atopic infants had increased *Clostridium* cells in their feces compared to non-atopic, healthy infants, concluding that increased *Clostridium* numbers were associated with childhood allergic sensitization and that differences in neonatal gut microbiome and bacterial fatty acids precede the development of atopy in infants (158). These data also suggest the key role that the microbiome and their metabolites play in the development and maturation of the immune system.

We have previously shown that oral supplementation of mice with *Lactobacillus johnsonii* resulted in significantly reduced airway allergic sensitization and RSV-induced pulmonary immunopathology (127). The gut microbiota was modified considerably in the supplemented mice, but *L. johnsonii* was not detected in the airways, and furthermore, only viable *L. johnsonii* was able to generate protection (127). Following this study, we tested the metabolic potential of *L. johnsonii* for its impact on the lung immune response against RSV, and we found that *L. johnsonii* supplementation of the mice diet modified the systemic metabolic profile. In plasma samples analyzed by liquid chromatography/mass spectrometry, we were able to detect more than 50 different metabolites altered at baseline in supplemented mice (128). Following RSV infection, the plasma samples from *L. johnsonii* supplemented animals exhibited substantial metabolic reprogramming in response to RSV infection, involving significant increases in a broad range of lipid-, bile-, amino acid, and peptide-derived metabolites (128). We also observed that RSV-infected bone-marrow derived DCs from *L. johnsonii* supplemented mice had altered cytokine secretion, reduced expression of co-stimulatory molecules, and modified CD4^+^ T cell cytokine production. In this study, we outlined a potential mechanism for immune alteration by manipulating the microbiome and facilitating the availability of a complex and broad profile of microbial and mammalian-derived immunomodulatory metabolites that collectively may play a regulatory role (128). This result supports the idea that the metabolic potential of the gut microbiome is substantial and extends beyond fatty acid production, and may extend to immune regulation at tissues other than the gut.

In a US birth cohort of neonates, stool samples were analyzed to identify differences in microbiota-composition and associated metabolites. The authors observed that the microbiome composition of the infants that developed multi-sensitized atopy at the age of 2 years, present significant different compositions of microbial communities than those who did not. The group of children who developed atopy showed lower relative abundance of specific bacteria (*Bifidobacterium, Akkermansia*, and *Faecalibacterium*) and higher relative abundance of fungi (*Candida* and *Rhodotorula*) (160). Furthermore, the authors observed a distinct set of fecal metabolites, which were tested to generate pro-inflammatory responses using sterile fecal water from the samples of the infants with higher risk of developing allergies. When adding this sterile fecal water to the growth medium of adult human peripheral T cells, the authors observed an increased proportion of CD4^+^ IL-4^+^ cells and reduced numbers of regulatory T cells (160). Interestingly, one of the metabolites identified in the fecal samples of the high-risk infants was 12,13-DiHOME, which can suppress Treg cells. Addition of 12,13-DiHOME recapitulated the effect of the fecal water on T cell culture. This suggests that the gut microbiome in infants can modify the metabolic profile and that those metabolites can potentially impact the immune response to cause the development of allergies in later life (160). This study correlates with another study in which bronchoalveolar lavage fluid (BALF) was collected from adult pollen-allergic patients with mild asthma (age 22–42 years) and healthy non-allergic people, in basal conditions or under inhaled-pollen challenged (161). 87 lipid mediators were screened, and a significant difference in lipid metabolites was observed between allergic and healthy patients, including increases in both 12,13-DiHOME and 9,10-DiHOME in the BALF of allergic patients (161). These data highlight the importance of metabolites in lung allergic disease through modulation of the immune response, and the impact of the microbiome on the metabolic profile of the host. These pioneering studies suggest a young field ripe for further scientific investigation bringing together the microbiome and its metabolites and exploring their relationship to a healthy immune response.

## Concluding Remarks

Severe RSV infection followed by the development of allergies and asthma is determined by the interaction of environmental and inherited factors (162, 163). Nevertheless, genetics cannot explain the continued increased in allergies and asthma, and any change in population genetics would require multiple generations to occur (162). On the other hand, epigenetics could be induced more rapidly and can be generated by environmental changes. Importantly epigenetic modifications can be passed down from parents to offspring or can result from *in utero* modification (162). As we describe above, epigenetic changes can modify the immune phenotype, with alterations inherited from the mother are fivefold more significant than the paternal factors (140, 164).

The ability of epigenetic modifications to impact gene expression to regulate or dysregulate the homeostasis of the organisms has been widely studied from cancer to modulation of the immune system (165). The concept of modulation of the immune response through epigenetic modification caused by environmental factors, diet, microbiome, and metabolites may have clinical therapeutic implications for human health (165). Future studies will now focus on how the microbiome metabolite profiles impact immune responses in the long term through epigenetic alteration of not only ongoing immune responses but also progenitor cell populations. These changes would have long acting effects on future immune responses. Whether these changes can be reversed will be a significant challenge for future therapeutic progress (165).

Many known factors that predispose to develop severe RSV disease are linked, from the immune system and lung development through microbiome composition (Figure 1). In this review, we summarize the role of diverse factors that can impact the immune response during RSV infection. The further pursuit of the interactions among the environment, the microbiome, and its metabolites, and their impact on epigenetics in the context of severe RSV infection has the potential to reveal new therapeutics to alter the development of this disease and the predisposition to asthma. Ideally, immune development would be altered favorably early in childhood prior to the initiation of pathogenic immune phenotypes including the development of severe disease caused by RSV infection, and this is likely to be a future focus of many studies. The complex interactions among the numerous factors reviewed herein will likely also have far reaching effects on other aspects and types of disease.

## Author Contributions

WF, NL, and CP conceived of and wrote the manuscript.

## Conflict of Interest Statement

The authors declare that the research was conducted in the absence of any commercial or financial relationships that could be construed as a potential conflict of interest.

## References

[1] Henderson J Hilliard TN Sherriff A Stalker D Al Shammari N Thomas HM. Hospitalization for RSV bronchiolitis before 12 months of age and subsequent asthma, atopy and wheeze: a longitudinal birth cohort study. Pediatr Allergy Immunol (2005) 16:386–92. 10.1111/j.1399-3038.2005.00298.x 16101930

[2] Hall CB Weinberg GA Iwane MK Blumkin AK Edwards KM Staat MA The burden of respiratory syncytial virus infection in young children. N Engl J Med (2009) 360:588–98. 10.1056/NEJMoa0804877 19196675 PMC4829966

[3] Mukherjee S Lindell DM Berlin AA Morris SB Shanley TP Hershenson MB IL-17-induced pulmonary pathogenesis during respiratory viral infection and exacerbation of allergic disease. Am J Pathol (2011) 179:248–58. 10.1016/j.ajpath.2011.03.003 21703407 PMC3123803

[4] de Almeida Nagata DE Demoor T Ptaschinski C Ting HA Jang S Reed M IL-27R-mediated regulation of IL-17 controls the development of respiratory syncytial virus-associated pathogenesis. Am J Pathol (2014) 184:1807–18. 10.1016/j.ajpath.2014.02.004 24726498 PMC4044717

[5] Openshaw PJ Dean GS Culley FJ. Links between respiratory syncytial virus bronchiolitis and childhood asthma: clinical and research approaches. Pediatr Infect Dis (2003) 22:S58–64. 10.1097/00006454-200302001-00009 12671454

[6] Reed M Morris SH Owczarczyk AB Lukacs NW. Deficiency of autophagy protein Map1-LC3b mediates IL-17-dependent lung pathology during respiratory viral infection via ER stress-associated IL-1. Mucosal Immunol (2015) 8:1118–30. 10.1038/mi.2015.3 25669150 PMC4532659

[7] Hu J Robinson JL. Treatment of respiratory syncytial virus with palivizumab: a systematic review. World J Pediatr (2010) 6:296–300. 10.1007/s12519-010-0230-z 21080142

[8] Fonseca W Ozawa M Hatta M Orozco E Martinez MB Kawaoka Y. A recombinant influenza virus vaccine expressing the F protein of respiratory syncytial virus. Arch Virol (2014) 159:1067–77. 10.1007/s00705-013-1932-z 24292020 PMC4013198

[9] Garcia CG Bhore R Soriano-Fallas A Trost M Chason R Ramilo O Risk factors in children hospitalized with RSV bronchiolitis versus non-RSV bronchiolitis. Pediatrics (2010) 126:e1453–60. 10.1542/peds.2010-0507 21098154 PMC3761792

[10] Adkins B Leclerc C Marshall-Clarke S Neonatal adaptive immunity comes of age. Nat Rev Immunol (2004) 4:553–64. 10.1038/nri1394 15229474

[11] Makhseed M Raghupathy R Azizieh F Omu A Al-Shamali E Ashkanani L. Th1 and Th2 cytokine profiles in recurrent aborters with successful pregnancy and with subsequent abortions. Hum Reprod (2001) 16:2219–26. 10.1093/humrep/16.10.2219 11574519

[12] Danis B George TC Goriely S Dutta B Renneson J Gatto L Interferon regulatory factor 7-mediated responses are defective in cord blood plasmacytoid dendritic cells. Eur J Immunol (2008) 38:507–17. 10.1002/eji.200737760 18200500

[13] Corbett NP Blimkie D Ho KC Cai B Sutherland DP Kallos A Ontogeny of toll-like receptor mediated cytokine responses of human blood mononuclear cells. PLoS One (2010) 5:e15041. 10.1371/journal.pone.0015041 21152080 PMC2994830

[14] Kollmann TR Crabtree J Rein-Weston A Blimkie D Thommai F Wang XY Neonatal innate TLR-mediated responses are distinct from those of adults. J Immunol (2009) 183:7150–60. 10.4049/jimmunol.0901481 19917677 PMC4556237

[15] Black A Bhaumik S Kirkman RL Weaver CT Randolph DA. Developmental regulation of Th17-cell capacity in human neonates. Eur J Immunol (2012) 42:311–9. 10.1002/eji.201141847 22101893 PMC3414367

[16] Levy O Coughlin M Cronstein BN Roy RM Desai A Wessels MR. The adenosine system selectively inhibits TLR-mediated TNF-alpha production in the human newborn. J Immunol (2006) 177:1956–66. 10.4049/jimmunol.177.3.1956 16849509 PMC2881468

[17] Link AA Kino T Worth JA McGuire JL Crane ML Chrousos GP Ligand-activation of the adenosine A2a receptors inhibits IL-12 production by human monocytes. J Immunol (2000) 164:436–42. 10.4049/jimmunol.164.1.436 10605040

[18] Hasko G Kuhel DG Chen JF Schwarzschild MA Deitch EA Mabley JG Adenosine inhibits IL-12 and TNF-[alpha] production via adenosine A2a receptor-dependent and independent mechanisms. FASEB J (2000) 14:2065–74. 10.1096/fj.99-0508com 11023991

[19] Hasko G Nemeth ZH Vizi ES Salzman AL Szabo C. An agonist of adenosine A3 receptors decreases interleukin-12 and interferon-gamma production and prevents lethality in endotoxemic mice. Eur J Pharmacol (1998) 358:261–8. 10.1016/S0014-2999(98)00619-0 9822893

[20] Encabo A Solves P Carbonell-Uberos F Minana MD. The functional immaturity of dendritic cells can be relevant to increased tolerance associated with cord blood transplantation. Transfusion (2007) 47:272–9. 10.1111/j.1537-2995.2007.01103.x 17302774

[21] De Wit D Tonon S Olislagers V Goriely S Boutriaux M Goldman M Impaired responses to toll-like receptor 4 and toll-like receptor 3 ligands in human cord blood. J Autoimmun (2003) 21:277–81. 10.1016/j.jaut.2003.08.003 14599853

[22] Hunt DW Huppertz HI Jiang HJ Petty RE. Studies of human cord blood dendritic cells: evidence for functional immaturity. Blood (1994) 84:4333–43. 7994049

[23] Borras FE Matthews NC Lowdell MW Navarrete CV. Identification of both myeloid CD11c+ and lymphoid CD11c- dendritic cell subsets in cord blood. Br J Haematol (2001) 113:925–31. 10.1046/j.1365-2141.2001.02840.x 11442485

[24] De Wit D Olislagers V Goriely S Vermeulen F Wagner H Goldman M Blood plasmacytoid dendritic cell responses to CpG oligodeoxynucleotides are impaired in human newborns. Blood (2004) 103:1030–2. 10.1182/blood-2003-04-1216 14504106

[25] Currie SM Findlay EG McHugh BJ Mackellar A Man T Macmillan D The human cathelicidin LL-37 has antiviral activity against respiratory syncytial virus. PLoS One (2013) 8:e73659. 10.1371/journal.pone.0073659 24023689 PMC3758310

[26] Jaovisidha P Peeples ME Brees AA Carpenter LR Moy JN. Respiratory syncytial virus stimulates neutrophil degranulation and chemokine release. J Immunol (1999) 163:2816–20. 10453026

[27] Beauvillain C Delneste Y Scotet M Peres A Gascan H Guermonprez P Neutrophils efficiently cross-prime naive T cells in vivo. Blood (2007) 110:2965–73. 10.1182/blood-2006-12-063826 17562875

[28] Cavarra E Martorana PA Gambelli F de Santi M van Even P Lungarella G. Neutrophil recruitment into the lungs is associated with increased lung elastase burden, decreased lung elastin, and emphysema in alpha 1 proteinase inhibitor-deficient mice. Lab Invest (1996) 75:273–80. 8765327

[29] Strunk T Temming P Gembruch U Reiss I Bucsky P Schultz C. Differential maturation of the innate immune response in human fetuses. Pediatr Res (2004) 56:219–26. 10.1203/01.PDR.0000132664.66975.79 15181184

[30] Christensen RD Rothstein G. Efficiency of neutrophil migration in the neonate. Pediatr Res (1980) 14:1147–9. 10.1203/00006450-198010000-00013 7465286

[31] Hashimoto Y Moki T Takizawa T Shiratsuchi A Nakanishi Y. Evidence for phagocytosis of influenza virus-infected, apoptotic cells by neutrophils and macrophages in mice. J Immunol (2007) 178:2448–57. 10.4049/jimmunol.178.4.2448 17277152

[32] Funchal GA Jaeger N Czepielewski RS Machado MS Muraro SP Stein RT Respiratory syncytial virus fusion protein promotes TLR-4-dependent neutrophil extracellular trap formation by human neutrophils. PLoS One (2015) 10:e0124082. 10.1371/journal.pone.0124082 25856628 PMC4391750

[33] Wang Y Xu H Zheng X Wei H Sun R Tian Z. High expression of NKG2A/CD94 and low expression of granzyme B are associated with reduced cord blood NK cell activity. Cell Mol Immunol (2007) 4:377–82. 17976318

[34] Le Garff-Tavernier M Beziat V Decocq J Siguret V Gandjbakhch F Pautas E Human NK cells display major phenotypic and functional changes over the life span. Aging Cell (2010) 9:527–35. 10.1111/j.1474-9726.2010.00584.x 20477761

[35] Ribeiro LZ Tripp RA Rossi LM Palma PV Yokosawa J Mantese OC Serum mannose-binding lectin levels are linked with respiratory syncytial virus (RSV) disease. J Clin Immunol (2008) 28:166–73. 10.1007/s10875-007-9141-8 17952574

[36] Lau AS Sigaroudinia M Yeung MC Kohl S. Interleukin-12 induces interferon-gamma expression and natural killer cytotoxicity in cord blood mononuclear cells. Pediatr Res (1996) 39:150–5. 10.1203/00006450-199601000-00023 8825401

[37] Bonnema JD Rivlin KA Ting AT Schoon RA Abraham RT Leibson PJ. Cytokine-enhanced NK cell-mediated cytotoxicity. Positive modulatory effects of IL-2 and IL-12 on stimulus-dependent granule exocytosis. J Immunol (1994) 152:2098–104. 7907631

[38] Nguyen QH Roberts RL Ank BJ Lin SJ Thomas EK Stiehm ER. Interleukin (IL)-15 enhances antibody-dependent cellular cytotoxicity and natural killer activity in neonatal cells. Cell Immunol (1998) 185:83–92. 10.1006/cimm.1998.1286 9636686

[39] Lindell DM Morris SB White MP Kallal LE Lundy PK Hamouda T A novel inactivated intranasal respiratory syncytial virus vaccine promotes viral clearance without Th2 associated vaccine-enhanced disease. PLoS One (2011) 6:e21823. 10.1371/journal.pone.0021823 21789184 PMC3137595

[40] Qureshi MH Garvy BA. Neonatal T cells in an adult lung environment are competent to resolve *Pneumocystis carinii* pneumonia. J Immunol (2001) 166:5704–11. 10.4049/jimmunol.166.9.5704 11313412

[41] Haines CJ Giffon TD Lu LS Lu X Tessier-Lavigne M Ross DT Human CD4+ T cell recent thymic emigrants are identified by protein tyrosine kinase 7 and have reduced immune function. J Exp Med (2009) 206:275–85. 10.1084/jem.20080996 19171767 PMC2646563

[42] Hayakawa S Ohno N Okada S Kobayashi M. Significant augmentation of regulatory T cell numbers occurs during the early neonatal period. Clin Exp Immunol (2017) 190:268–79. 10.1111/cei.13008 28677152 PMC5629449

[43] Fan H Yang J Hao J Ren Y Chen L Li G Comparative study of regulatory T cells expanded ex vivo from cord blood and adult peripheral blood. Immunology (2012) 136:218–30. 10.1111/j.1365-2567.2012.03573.x 22348606 PMC3403260

[44] Rabe H Lundell AC Andersson K Adlerberth I Wold AE Rudin A. Higher proportions of circulating FOXP3+ and CTLA-4+ regulatory T cells are associated with lower fractions of memory CD4+ T cells in infants. J Leukoc Biol (2011) 90:1133–40. 10.1189/jlb.0511244 21934066 PMC3236549

[45] Mold JE Michaelsson J Burt TD Muench MO Beckerman KP Busch MP Maternal alloantigens promote the development of tolerogenic fetal regulatory T cells in utero. Science (2008) 322:1562–5. 10.1126/science.1164511 19056990 PMC2648820

[46] Brandenburg AH Groen J van Steensel-Moll HA Claas EC Rothbarth PH Neijens HJ Respiratory syncytial virus specific serum antibodies in infants under six months of age: limited serological response upon infection. J Med Virol (1997) 52:97–104. 10.1002/(SICI)1096-9071(199705)52:1<97::AID-JMV16>3.0.CO;2-Y 9131465

[47] Samukawa T Yamanaka N Hollingshead S Klingman K Faden H. Immune responses to specific antigens of *Streptococcus pneumoniae* and *Moraxella catarrhalis* in the respiratory tract. Infect Immun (2000) 68:1569–73. 10.1128/IAI.68.3.1569-1573.2000 10678976 PMC97317

[48] Plebani A Ugazio AG Avanzini MA Massimi P Zonta L Monafo V Serum IgG subclass concentrations in healthy subjects at different age: age normal percentile charts. Eur J Pediatr (1989) 149:164–7. 10.1007/BF01958271 2515060

[49] Schroeder HW Jr Mortari F Shiokawa S Kirkham PM Elgavish RA Bertrand FE III. Developmental regulation of the human antibody repertoire. Ann N Y Acad Sci (1995) 764:242–60. 10.1111/j.1749-6632.1995.tb55834.x 7486531

[50] Ridings J Nicholson IC Goldsworthy W Haslam R Roberton DM Zola H. Somatic hypermutation of immunoglobulin genes in human neonates. Clin Exp Immunol (1997) 108:366–74. 10.1046/j.1365-2249.1997.3631264.x 9158112 PMC1904651

[51] Ridings J Dinan L Williams R Roberton D Zola H. Somatic mutation of immunoglobulin V(H)6 genes in human infants. Clin Exp Immunol (1998) 114:33–9. 10.1046/j.1365-2249.1998.00694.x 9764600 PMC1905087

[52] Munoz FM. Respiratory syncytial virus in infants: is maternal vaccination a realistic strategy? Curr Opin Infect Dis (2015) 28:221–4. 10.1097/QCO.0000000000000161 25918956

[53] August A Glenn GM Kpamegan E Hickman SP Jani D Lu H A phase 2 randomized, observer-blind, placebo-controlled, dose-ranging trial of aluminum-adjuvanted respiratory syncytial virus F particle vaccine formulations in healthy women of childbearing age. Vaccine (2017) 35:3749–59. 10.1016/j.vaccine.2017.05.045 28579233

[54] Garg R Latimer L Wang Y Simko E Gerdts V Potter A Maternal immunization with respiratory syncytial virus fusion protein formulated with a novel combination adjuvant provides protection from RSV in newborn lambs. Vaccine (2016) 34:261–9. 10.1016/j.vaccine.2015.11.029 26616551

[55] Doering G Gusenleitner W Belohradsky BH Burdach S Resch B Liese JG. The risk of respiratory syncytial virus-related hospitalizations in preterm infants of 29 to 35 weeks’ gestational age. Pediatr Infect Dis J (2006) 25:1188–90. 10.1097/01.inf.0000246978.58565.b5 17133170

[56] Weisman L. Populations at risk for developing respiratory syncytial virus and risk factors for respiratory syncytial virus severity: infants with predisposing conditions. Pediatr Infect Dis J (2003) 22:S33–7. 10.1097/01.inf.0000053883.08663.e5 12671450

[57] Simister NE. Placental transport of immunoglobulin G. Vaccine (2003) 21:3365–9. 10.1016/S0264-410X(03)00334-7 12850341

[58] Englund JA. Passive protection against respiratory syncytial virus disease in infants: the role of maternal antibody. Pediatr Infect Dis J (1994) 13:449–53. 10.1097/00006454-199405000-00037 8072835

[59] Sacchi F Rondini G Mingrat G Stronati M Gancia GP Marseglia GL Different maturation of neutrophil chemotaxis in term and preterm newborn infants. J Pediatr (1982) 101:273–4. 10.1016/S0022-3476(82)80139-X 7097428

[60] Carr R Pumford D Davies JM. Neutrophil chemotaxis and adhesion in preterm babies. Arch Dis Child (1992) 67:813–7. 10.1136/adc.67.7_Spec_No.813 1519981 PMC1590408

[61] Nupponen I Turunen R Nevalainen T Peuravuori H Pohjavuori M Repo H Extracellular release of bactericidal/permeability-increasing protein in newborn infants. Pediatr Res (2002) 51:670–4. 10.1203/00006450-200206000-00002 12032259

[62] Raymond SL Mathias BJ Murphy TJ Rincon JC Lopez MC Ungaro R Neutrophil chemotaxis and transcriptomics in term and preterm neonates. Transl Res (2017) 190:4–15. 10.1016/j.trsl.2017.08.003 28873345 PMC5705589

[63] Assefa D Amin N Dozor AJ Parton LA. Attenuated interleukin-8/leukocyte immunoresponse in preterm infants compared with term infants hospitalized with respiratory syncytial virus bronchiolitis: a pilot study. Hum Immunol (2011) 72:708–11. 10.1016/j.humimm.2011.05.016 21683109

[64] Kerrin A Fitch P Errington C Kerr D Waxman L Riding K Differential lower airway dendritic cell patterns may reveal distinct endotypes of RSV bronchiolitis. Thorax (2017) 72:620–7. 10.1136/thoraxjnl-2015-207358 27531529

[65] Bermick JR Lambrecht NJ denDekker AD Kunkel SL Lukacs NW Hogaboam CM Neonatal monocytes exhibit a unique histone modification landscape. Clin Epigenetics (2016) 8:99. 10.1186/s13148-016-0265-7 27660665 PMC5028999

[66] Marr N Wang TI Kam SH Hu YS Sharma AA Lam A Attenuation of respiratory syncytial virus-induced and RIG-I-dependent type I IFN responses in human neonates and very young children. J Immunol (2014) 192:948–57. 10.4049/jimmunol.1302007 24391215

[67] Morrisey EE Cardoso WV Lane RH Rabinovitch M Abman SH Ai X Molecular determinants of lung development. Ann Am Thorac Soc (2013) 10:S12–6. 10.1513/AnnalsATS.201207-036OT 23607856 PMC3955361

[68] Morrisey EE Hogan BL. Preparing for the first breath: genetic and cellular mechanisms in lung development. Dev Cell (2010) 18:8–23. 10.1016/j.devcel.2009.12.010 20152174 PMC3736813

[69] Madurga A Mizíková I Ruiz-Camp J Morty RE. Recent advances in late lung development and the pathogenesis of bronchopulmonary dysplasia. Am J Physiol Lung Cell Mol Physiol (2013) 305:L893–905. 10.1152/ajplung.00267.2013 24213917

[70] Boyce TG Mellen BG Mitchel EF Jr Wright PF Griffin MR. Rates of hospitalization for respiratory syncytial virus infection among children in medicaid. J Pediatr (2000) 137:865–70. 10.1067/mpd.2000.110531 11113845

[71] Koch CM Andrews RM Flicek P Dillon SC Karaoz U Clelland GK The landscape of histone modifications across 1% of the human genome in five human cell lines. Genome Res (2007) 17:691–707. 10.1101/gr.5704207 17567990 PMC1891331

[72] Pekowska A Benoukraf T Zacarias-Cabeza J Belhocine M Koch F Holota H H3K4 tri-methylation provides an epigenetic signature of active enhancers. EMBO J (2011) 30:4198–210. 10.1038/emboj.2011.295 21847099 PMC3199384

[73] Riising EM Comet I Leblanc B Wu X Johansen JV Helin K. Gene silencing triggers polycomb repressive complex 2 recruitment to CpG islands genome wide. Mol Cell (2014) 55:347–60. 10.1016/j.molcel.2014.06.005 24999238

[74] Okano M Bell DW Haber DA Li E. DNA methyltransferases Dnmt3a and Dnmt3b are essential for de novo methylation and mammalian development. Cell (1999) 99:247–57. 10.1016/S0092-8674(00)81656-6 10555141

[75] Macleod D Charlton J Mullins J Bird AP. Sp1 sites in the mouse aprt gene promoter are required to prevent methylation of the CpG Island. Genes Dev (1994) 8:2282–92. 10.1101/gad.8.19.2282 7958895

[76] Siegfried Z Eden S Mendelsohn M Feng X Tsuberi BZ Cedar H. DNA methylation represses transcription in vivo. Nat Genet (1999) 22:203–6. 10.1038/9727 10369268

[77] Deaton AM Bird A. CpG islands and the regulation of transcription. Genes Dev (2011) 25:1010–22. 10.1101/gad.2037511 21576262 PMC3093116

[78] Lim LP Lau NC Garrett-Engele P Grimson A Schelter JM Castle J Microarray analysis shows that some microRNAs downregulate large numbers of target mRNAs. Nature (2005) 433:769–73. 10.1038/nature03315 15685193

[79] Lai EC. Micro RNAs are complementary to 3’ UTR sequence motifs that mediate negative post-transcriptional regulation. Nat Genet (2002) 30:363–4. 10.1038/ng865 11896390

[80] Petersen CP Bordeleau ME Pelletier J Sharp PA. Short RNAs repress translation after initiation in mammalian cells. Mol Cell (2006) 21:533–42. 10.1016/j.molcel.2006.01.031 16483934

[81] Lee GR Kim ST Spilianakis CG Fields PE Flavell RA. T helper cell differentiation: regulation by cis elements and epigenetics. Immunity (2006) 24:369–79. 10.1016/j.immuni.2006.03.007 16618596

[82] Wei G Wei L Zhu J Zang C Hu-Li J Yao Z Global mapping of H3K4me3 and H3K27me3 reveals specificity and plasticity in lineage fate determination of differentiating CD4+ T cells. Immunity (2009) 30:155–67. 10.1016/j.immuni.2008.12.009 19144320 PMC2722509

[83] Wen H Dou Y Hogaboam CM Kunkel SL. Epigenetic regulation of dendritic cell-derived interleukin-12 facilitates immunosuppression after a severe innate immune response. Blood (2008) 111:1797–804. 10.1182/blood-2007-08-106443 18055863 PMC2234040

[84] Lee DU Agarwal S Rao A. Th2 lineage commitment and efficient IL-4 production involves extended demethylation of the IL-4 gene. Immunity (2002) 16:649–60. 10.1016/S1074-7613(02)00314-X 12049717

[85] Schoenborn JR Dorschner MO Sekimata M Santer DM Shnyreva M Fitzpatrick DR Comprehensive epigenetic profiling identifies multiple distal regulatory elements directing transcription of the gene encoding interferon-gamma. Nat Immunol (2007) 8:732–42. 10.1038/ni0807-893b 17546033 PMC2144744

[86] Rose S Lichtenheld M Foote MR Adkins B. Murine neonatal CD4+ cells are poised for rapid Th2 effector-like function. J Immunol (2007) 178:2667–78. 10.4049/jimmunol.178.5.2667 17312108 PMC2112939

[87] Webster RB Rodriguez Y Klimecki WT Vercelli D. The human IL-13 locus in neonatal CD4+ T cells is refractory to the acquisition of a repressive chromatin architecture. J Biol Chem (2007) 282:700–9. 10.1074/jbc.M609501200 17090525

[88] Martino D Maksimovic J Joo JH Prescott SL Saffery R. Genome-scale profiling reveals a subset of genes regulated by DNA methylation that program somatic T-cell phenotypes in humans. Genes Immun (2012) 13:388–98. 10.1038/gene.2012.7 22495533

[89] Martino DJ Tulic MK Gordon L Hodder M Richman TR Metcalfe J Evidence for age-related and individual-specific changes in DNA methylation profile of mononuclear cells during early immune development in humans. Epigenetics (2011) 6:1085–94. 10.4161/epi.6.9.16401 21814035

[90] White GP Hollams EM Yerkovich ST Bosco A Holt BJ Bassami MR CpG methylation patterns in the IFNgamma promoter in naive T cells: variations during Th1 and Th2 differentiation and between atopics and non-atopics. Pediatr Allergy Immunol (2006) 17:557–64. 10.1111/j.1399-3038.2006.00465.x 17121582

[91] White GP Watt PM Holt BJ Holt PG. Differential patterns of methylation of the IFN-gamma promoter at CpG and non-CpG sites underlie differences in IFN-gamma gene expression between human neonatal and adult CD45RO- T cells. J Immunol (2002) 168:2820–7. 10.4049/jimmunol.168.6.2820 11884451

[92] Jacoby M Gohrbandt S Clausse V Brons NH Muller CP. Interindividual variability and co-regulation of DNA methylation differ among blood cell populations. Epigenetics (2012) 7:1421–34. 10.4161/epi.22845 23151460 PMC3528697

[93] Merkerova M Vasikova A Belickova M Bruchova H. MicroRNA expression profiles in umbilical cord blood cell lineages. Stem Cells Dev (2010) 19:17–26. 10.1089/scd.2009.0071 19435428

[94] Yu HR Hsu TY Huang HC Kuo HC Li SC Yang KD Comparison of the functional microRNA expression in immune cell subsets of neonates and adults. Front Immunol (2016) 7:615. 10.3389/fimmu.2016.00615 28066425 PMC5165026

[95] Lederhuber H Baer K Altiok I Sadeghi K Herkner KR Kasper DC. MicroRNA-146: tiny player in neonatal innate immunity? Neonatology (2011) 99:51–6. 10.1159/000301938 20616571

[96] Taganov KD Boldin MP Chang KJ Baltimore D. NF-kappaB-dependent induction of microRNA miR-146, an inhibitor targeted to signaling proteins of innate immune responses. Proc Natl Acad Sci U S A (2006) 103:12481–6. 10.1073/pnas.0605298103 16885212 PMC1567904

[97] Wissink EM Smith NL Spektor R Rudd BD Grimson A. MicroRNAs and their targets are differentially regulated in adult and neonatal mouse CD8+ T cells. Genetics (2015) 201:1017–30. 10.1534/genetics.115.179176 26416483 PMC4649632

[98] Palin AC Ramachandran V Acharya S Lewis DB. Human neonatal naive CD4+ T cells have enhanced activation-dependent signaling regulated by the microRNA miR-181a. J Immunol (2013) 190:2682–91. 10.4049/jimmunol.1202534 23408835 PMC3952015

[99] Weitzel RP Lesniewski ML Haviernik P Kadereit S Leahy P Greco NJ microRNA 184 regulates expression of NFAT1 in umbilical cord blood CD4+ T cells. Blood (2009) 113:6648–57. 10.1182/blood-2008-09-181156 19286996 PMC2710921

[100] Goriely S Van Lint C Dadkhah R Libin M De Wit D Demonte D A defect in nucleosome remodeling prevents IL-12(p35) gene transcription in neonatal dendritic cells. J Exp Med (2004) 199:1011–6. 10.1084/jem.20031272 15051764 PMC2211877

[101] Porras A Kozar S Russanova V Salpea P Hirai T Sammons N Developmental and epigenetic regulation of the human TLR3 gene. Mol Immunol (2008) 46:27–36. 10.1016/j.molimm.2008.06.030 18715647 PMC6287275

[102] Stevenson MD Mansbach JM Mowad E Dunn M Clark S Piedra PA Prenatal versus postnatal tobacco smoke exposure and intensive care use in children hospitalized with bronchiolitis. Acad Pediatr (2016) 16:446–52. 10.1016/j.acap.2015.11.001 26555856 PMC4871768

[103] Breton CV Byun HM Wenten M Pan F Yang A Gilliland FD. Prenatal tobacco smoke exposure affects global and gene-specific DNA methylation. Am J Respir Crit Care Med (2009) 180:462–7. 10.1164/rccm.200901-0135OC 19498054 PMC2742762

[104] Rotroff DM Joubert BR Marvel SW Haberg SE Wu MC Nilsen RM Maternal smoking impacts key biological pathways in newborns through epigenetic modification in Utero. BMC Genomics (2016) 17:976. 10.1186/s12864-016-3310-1 27887572 PMC5124223

[105] Singh SP Chand HS Langley RJ Mishra N Barrett T Rudolph K Gestational exposure to sidestream (secondhand) cigarette smoke promotes transgenerational epigenetic transmission of exacerbated allergic asthma and bronchopulmonary dysplasia. J Immunol (2017) 198:3815–22. 10.4049/jimmunol.1700014 28381639 PMC5473031

[106] Pauwels S Ghosh M Duca RC Bekaert B Freson K Huybrechts I Dietary and supplemental maternal methyl-group donor intake and cord blood DNA methylation. Epigenetics (2017) 12:1–10. 10.1080/15592294.2016.1257450 27830979 PMC5270634

[107] Haberg SE London SJ Stigum H Nafstad P Nystad W. Folic acid supplements in pregnancy and early childhood respiratory health. Arch Dis Child (2009) 94:180–4. 10.1136/adc.2008.142448 19052032 PMC3612898

[108] Whitrow MJ Moore VM Rumbold AR Davies MJ. Effect of supplemental folic acid in pregnancy on childhood asthma: a prospective birth cohort study. Am J Epidemiol (2009) 170:1486–93. 10.1093/aje/kwp315 19880541

[109] Kroetz DN Allen RM Schaller MA Cavallaro C Ito T Kunkel SL. Type I interferon induced epigenetic regulation of macrophages suppresses innate and adaptive immunity in acute respiratory viral infection. PLoS Pathog (2015) 11:e1005338. 10.1371/journal.ppat.1005338 26709698 PMC4692439

[110] Ptaschinski C Mukherjee S Moore ML Albert M Helin K Kunkel SL RSV-induced H3K4 demethylase KDM5B leads to regulation of dendritic cell-derived innate cytokines and exacerbates pathogenesis in vivo. PLoS Pathog (2015) 11:e1004978. 10.1371/journal.ppat.1004978 26083387 PMC4470918

[111] Nagata DE Ting HA Cavassani KA Schaller MA Mukherjee S Ptaschinski C Epigenetic control of Foxp3 by SMYD3 H3K4 histone methyltransferase controls iTreg development and regulates pathogenic T-cell responses during pulmonary viral infection. Mucosal Immunol (2015) 8:1131–43. 10.1038/mi.2015.4 25669152 PMC4532649

[112] Spalluto CM Singhania A Cellura D Woelk CH Sanchez-Elsner T Staples KJ IFNgamma influences epithelial anti-viral responses via histone methylation of the rig-I promoter. Am J Respir Cell Mol Biol (2017) 57(4):428–38. 10.1165/rcmb.2016-0392OC 28481620

[113] Wu J Chen ZJ. Innate immune sensing and signaling of cytosolic nucleic acids. Annu Rev Immunol (2014) 32:461–88. 10.1146/annurev-immunol-032713-120156 24655297

[114] Feng Q Su Z Song S Chiu H Zhang B Yi L Histone deacetylase inhibitors suppress RSV infection and alleviate virus-induced airway inflammation. Int J Mol Med (2016) 38:812–22. 10.3892/ijmm.2016.2691 27460781 PMC4990302

[115] Wang L Wu G Qin X Ma Q Zhou Y Liu S Expression of nodal on bronchial epithelial cells influenced by lung microbes through DNA methylation modulates the differentiation of T-helper cells. Cell Physiol Biochem (2015) 37:2012–22. 10.1159/000438561 26584299

[116] Elgizouli M Logan C Nieters A Brenner H Rothenbacher D. Cord blood PRF1 methylation patterns and risk of lower respiratory tract infections in infants: findings from the Ulm birth cohort. Medicine (2015) 94:e332. 10.1097/MD.0000000000000332 25569648 PMC4602833

[117] Elgizouli M Logan C Grychtol R Rothenbacher D Nieters A Heinzmann A. Reduced PRF1 enhancer methylation in children with a history of severe RSV bronchiolitis in infancy: an association study. BMC Pediatr (2017) 17:65. 10.1186/s12887-017-0817-9 28253869 PMC5335730

[118] Kim RY Horvat JC Pinkerton JW Starkey MR Essilfie AT Mayall JR MicroRNA-21 drives severe, steroid-insensitive experimental asthma by amplifying phosphoinositide 3-kinase-mediated suppression of histone deacetylase 2. J Allergy Clin Immunol (2017) 139:519–32. 10.1016/j.jaci.2016.04.038 27448447

[119] Collison A Siegle JS Hansbro NG Kwok CT Herbert C Mattes J Epigenetic changes associated with disease progression in a mouse model of childhood allergic asthma. Dis Model Mech (2013) 6:993–1000. 10.1242/dmm.011247 23611895 PMC3701218

[120] Feldman AS He Y Moore ML Hershenson MB Hartert TV. Toward primary prevention of asthma. Reviewing the evidence for early-life respiratory viral infections as modifiable risk factors to prevent childhood asthma. Am J Respir Crit Care Med (2015) 191:34–44. 10.1164/rccm.201405-0901PP 25369458 PMC4299628

[121] Lynch JP Sikder MA Curren BF Werder RB Simpson J Cuiv PO The influence of the microbiome on early-life severe viral lower respiratory infections and asthma-food for thought? Front Immunol (2017) 8:156. 10.3389/fimmu.2017.00156 28261214 PMC5311067

[122] Simoes EA. Environmental and demographic risk factors for respiratory syncytial virus lower respiratory tract disease. J Pediatr (2003) 143:S118–26. 10.1067/S0022-3476(03)00511-0 14615710

[123] Kristensen K Fisker N Haerskjold A Ravn H Simoes EA Stensballe L. Caesarean section and hospitalization for respiratory syncytial virus infection: a population-based study. Pediatr Infect Dis J (2015) 34:145–8. 10.1097/INF.0000000000000552 25232778

[124] Vissers M de Groot R Ferwerda G. Severe viral respiratory infections: are bugs bugging? Mucosal Immunol (2014) 7:227–38. 10.1038/mi.2013.93 24220300

[125] Rodriguez JM Murphy K Stanton C Ross RP Kober OI Juge N The composition of the gut microbiota throughout life, with an emphasis on early life. Microb Ecol Health Dis (2015) 26:26050. 10.3402/mehd.v26.26050 25651996 PMC4315782

[126] Fujimura KE Johnson CC Ownby DR Cox MJ Brodie EL Havstad SL Man’s best friend? The effect of pet ownership on house dust microbial communities. J Allergy Clin Immunol (2010) 126:410–2. 10.1016/j.jaci.2010.05.042 20633927 PMC2956425

[127] Fujimura KE Demoor T Rauch M Faruqi AA Jang S Johnson CC House dust exposure mediates gut microbiome *Lactobacillus* enrichment and airway immune defense against allergens and virus infection. Proc Natl Acad Sci U S A (2014) 111:805–10. 10.1073/pnas.1310750111 24344318 PMC3896155

[128] Fonseca W Lucey K Jang S Fujimura KE Rasky A Ting HA *Lactobacillus johnsonii* supplementation attenuates respiratory viral infection via metabolic reprogramming and immune cell modulation. Mucosal Immunol (2017) 10(6):1569–80. 10.1038/mi.2017.13 28295020 PMC5599307

[129] Aagaard K Ma J Antony KM Ganu R Petrosino J Versalovic J. The placenta harbors a unique microbiome. Sci Transl Med (2014) 6:237ra265. 10.1126/scitranslmed.3008599 24848255 PMC4929217

[130] Rautava S Kainonen E Salminen S Isolauri E Maternal probiotic supplementation during pregnancy and breast-feeding reduces the risk of eczema in the infant. J Allergy Clin Immunol (2012) 130:1355–60. 10.1016/j.jaci.2012.09.003 23083673

[131] Fujimura KE Lynch SV. Microbiota in allergy and asthma and the emerging relationship with the gut microbiome. Cell Host Microbe (2015) 17:592–602. 10.1016/j.chom.2015.04.007 25974301 PMC4443817

[132] Rautava S Kalliomaki M Isolauri E. Probiotics during pregnancy and breast-feeding might confer immunomodulatory protection against atopic disease in the infant. J Allergy Clin Immunol (2002) 109:119–21. 10.1067/mai.2002.120273 11799376

[133] Stout MJ Zhou Y Wylie KM Tarr PI Macones GA Tuuli MG Early pregnancy vaginal microbiome trends and preterm birth. Am J Obstet Gynecol (2017) 217:e1–356. 10.1016/j.ajog.2017.05.030 PMC5581228 28549981

[134] Havstad S Wegienka G Zoratti EM Lynch SV Boushey HA Nicholas C Effect of prenatal indoor pet exposure on the trajectory of total IgE levels in early childhood. J Allergy Clin Immunol (2011) 128:880–885.e884. 10.1016/j.jaci.2011.06.039 21820714 PMC3185205

[135] Tochitani S Ikeno T Ito T Sakurai A Yamauchi T Matsuzaki H. Administration of non-absorbable antibiotics to pregnant mice to perturb the maternal gut microbiota is associated with alterations in offspring behavior. PLoS One (2016) 11:e0138293. 10.1371/journal.pone.0138293 26789865 PMC4720425

[136] Abrahamsson TR Jakobsson T Bottcher MF Fredrikson M Jenmalm MC Bjorksten B Probiotics in prevention of IgE-associated eczema: a double-blind, randomized, placebo-controlled trial. J Allergy Clin Immunol (2007) 119:1174–80. 10.1016/j.jaci.2007.01.007 17349686

[137] Kennedy EA Connolly J Hourihane JO Fallon PG McLean WH Murray D Skin microbiome before development of atopic dermatitis: early colonization with commensal *Staphylococci* at 2 months is associated with a lower risk of atopic dermatitis at 1 year. J Allergy Clin Immunol (2017) 139:166–72. 10.1016/j.jaci.2016.07.029 27609659 PMC5207796

[138] Arrieta MC Stiemsma LT Dimitriu PA Thorson L Russell S Yurist-Doutsch S Early infancy microbial and metabolic alterations affect risk of childhood asthma. Sci Transl Med (2015) 7:307ra152. 10.1126/scitranslmed.aab2271 26424567

[139] Levin AM Sitarik AR Havstad SL Fujimura KE Wegienka G Cassidy-Bushrow AE Joint effects of pregnancy, sociocultural, and environmental factors on early life gut microbiome structure and diversity. Sci Rep (2016) 6:31775. 10.1038/srep31775 27558272 PMC4997337

[140] Cookson WO Young RP Sandford AJ Moffatt MF Shirakawa T Sharp PA Maternal inheritance of atopic IgE responsiveness on chromosome 11q. Lancet (1992) 340:381–4. 10.1016/0140-6736(92)91468-N 1353553

[141] Hakansson S Kallen K. Caesarean section increases the risk of hospital care in childhood for asthma and gastroenteritis. Clin Exp Allergy (2003) 33:757–64. 10.1046/j.1365-2222.2003.01667.x 12801309

[142] Neu J Rushing J. Cesarean versus vaginal delivery: long-term infant outcomes and the hygiene hypothesis. Clin Perinatol (2011) 38:321–31. 10.1016/j.clp.2011.03.008 21645799 PMC3110651

[143] Bach JF The effect of infections on susceptibility to autoimmune and allergic diseases. N Engl J Med (2002) 347:911–20. 10.1056/NEJMra020100 12239261

[144] Meehan CJ Beiko RG. A phylogenomic view of ecological specialization in the Lachnospiraceae, a family of digestive tract-associated bacteria. Genome Biol Evol (2014) 6:703–13. 10.1093/gbe/evu050 24625961 PMC3971600

[145] Yasmin F Tun HM Konya TB Guttman DS Chari RS Field CJ Cesarean section, formula feeding, and infant antibiotic exposure: separate and combined impacts on gut microbial changes in later infancy. Front Pediatr (2017) 5:200. 10.3389/fped.2017.00200 29018787 PMC5622971

[146] Munblit D Verhasselt V. Allergy prevention by breastfeeding: possible mechanisms and evidence from human cohorts. Curr Opin Allergy Clin Immunol (2016) 16:427–33. 10.1097/ACI.0000000000000303 27518839

[147] Nowak-Wegrzyn A Chatchatee P. Mechanisms of tolerance induction. Ann Nutr Metab (2017) 70(Suppl 2):7–24. 10.1159/000457915 28521317

[148] Lanari M Prinelli F Adorni F Di Santo S Faldella G Silvestri M Maternal milk protects infants against bronchiolitis during the first year of life. Results from an Italian cohort of newborns. Early Hum Dev (2013) 89(Suppl 1):S51–7. 10.1016/S0378-3782(13)70016-1 23809352

[149] Nishimura T Suzue J Kaji H. Breastfeeding reduces the severity of respiratory syncytial virus infection among young infants: a multi-center prospective study. Pediatr Int (2009) 51:812–6. 10.1111/j.1442-200X.2009.02877.x 19419530

[150] Lanari M Prinelli F Adorni F Di Santo S Vandini S Silvestri M Risk factors for bronchiolitis hospitalization during the first year of life in a multicenter Italian birth cohort. Ital J Pediatr (2015) 41:40. 10.1186/s13052-015-0149-z 26006025 PMC4453833

[151] Huffnagle GB Dickson RP Lukacs NW. The respiratory tract microbiome and lung inflammation: a two-way street. Mucosal Immunol (2017) 10:299–306. 10.1038/mi.2016.108 27966551 PMC5765541

[152] Tomosada Y Chiba E Zelaya H Takahashi T Tsukida K Kitazawa H Nasally administered *Lactobacillus rhamnosus* strains differentially modulate respiratory antiviral immune responses and induce protection against respiratory syncytial virus infection. BMC Immunol (2013) 14:40. 10.1186/1471-2172-14-40 23947615 PMC3751766

[153] Chiba E Tomosada Y Vizoso-Pinto MG Salva S Takahashi T Tsukida K Immunobiotic *Lactobacillus rhamnosus* improves resistance of infant mice against respiratory syncytial virus infection. Int Immunopharmacol (2013) 17:373–82. 10.1016/j.intimp.2013.06.024 23838113

[154] Luoto R Ruuskanen O Waris M Kalliomaki M Salminen S Isolauri E. Prebiotic and probiotic supplementation prevents rhinovirus infections in preterm infants: a randomized, placebo-controlled trial. J Allergy Clin Immunol (2014) 133:405–13. 10.1016/j.jaci.2013.08.020 24131826 PMC7112326

[155] Riedler J Braun-Fahrlander C Eder W Schreuer M Waser M Maisch S Exposure to farming in early life and development of asthma and allergy: a cross-sectional survey. Lancet (2001) 358:1129–33. 10.1016/S0140-6736(01)06252-3 11597666

[156] Ownby DR Johnson CC Peterson EL. Exposure to dogs and cats in the first year of life and risk of allergic sensitization at 6 to 7 years of age. JAMA (2002) 288:963–72. 10.1001/jama.288.8.963 12190366

[157] Trompette A Gollwitzer ES Yadava K Sichelstiel AK Sprenger N Ngom-Bru C Gut microbiota metabolism of dietary fiber influences allergic airway disease and hematopoiesis. Nat Med (2014) 20:159–66. 10.1038/nm.3444 24390308

[158] Kalliomaki M Kirjavainen P Eerola E Kero P Salminen S Isolauri E. Distinct patterns of neonatal gut microflora in infants in whom atopy was and was not developing. J Allergy Clin Immunol (2001) 107:129–34. 10.1067/mai.2001.111237 11150002

[159] Sharon G Garg N Debelius J Knight R Dorrestein PC Mazmanian SK. Specialized metabolites from the microbiome in health and disease. Cell Metab (2014) 20:719–30. 10.1016/j.cmet.2014.10.016 25440054 PMC4337795

[160] Fujimura KE Sitarik AR Havstad S Lin DL Levan S Fadrosh D Neonatal gut microbiota associates with childhood multisensitized atopy and T cell differentiation. Nat Med (2016) 22:1187–91. 10.1038/nm.4176 27618652 PMC5053876

[161] Lundstrom SL Yang J Kallberg HJ Thunberg S Gafvelin G Haeggstrom JZ Allergic asthmatics show divergent lipid mediator profiles from healthy controls both at baseline and following birch pollen provocation. PLoS One (2012) 7:e33780. 10.1371/journal.pone.0033780 22438998 PMC3305349

[162] Begin P Nadeau KC. Epigenetic regulation of asthma and allergic disease. Allergy Asthma Clin Immunol (2014) 10:27. 10.1186/1710-1492-10-27 24932182 PMC4057652

[163] Palmer LJ Burton PR Faux JA James AL Musk AW Cookson WO. Independent inheritance of serum immunoglobulin E concentrations and airway responsiveness. Am J Respir Crit Care Med (2000) 161:1836–43. 10.1164/ajrccm.161.6.9805104 10852754

[164] Litonjua AA Carey VJ Burge HA Weiss ST Gold DR. Parental history and the risk for childhood asthma. Does mother confer more risk than father? Am J Respir Crit Care Med (1998) 158:176–81. 10.1164/ajrccm.158.1.9710014 9655726

[165] Berni Canani R Di Costanzo M Leone L. The epigenetic effects of butyrate: potential therapeutic implications for clinical practice. Clin Epigenetics (2012) 4:4. 10.1186/1868-7083-4-4 22414433 PMC3312834

